# A pragmatic study exploring the prevention of delirium among hospitalized older hip fracture patients: Applying evidence to routine clinical practice using clinical decision support

**DOI:** 10.1186/1748-5908-5-81

**Published:** 2010-10-22

**Authors:** Jayna M Holroyd-Leduc, Greg A Abelseth, Farah Khandwala, James L Silvius, David B Hogan, Heidi N Schmaltz, Cyril B Frank, Sharon E Straus

**Affiliations:** 1 Foothills Medical Center, 1403-29th Street NW, Calgary, University of Calgary, Calgary, Alberta, Canada; 2 University of Toronto, Toronto, Ontario, Canada

## Abstract

Delirium occurs in up to 65% of older hip fracture patients. Developing delirium in hospital has been associated with a variety of adverse outcomes. Trials have shown that multi-component preventive interventions can lower delirium rates. The objective of this study was to implement and evaluate the effectiveness of an evidence-based electronic care pathway, which incorporates multi-component delirium strategies, among older hip fracture patients. We conducted a pragmatic study using an interrupted time series design in order to evaluate the use and impact of the intervention. The target population was all consenting patients aged 65 years or older admitted with an acute hip fracture to the orthopedic units at two Calgary, Alberta hospitals. The primary outcome was delirium rates. Secondary outcomes included length of hospital stay, in-hospital falls, in-hospital mortality, new discharges to long-term care, and readmissions. A Durbin Watson test was conducted to test for serial correlation and, because no correlation was found, Chi-square statistics, Wilcoxon test and logistic regression analyses were conducted as appropriate. At study completion, focus groups were conducted at each hospital to explore issues around the use of the order set. During the 40-week study period, 134 patients were enrolled. The intervention had no effect on the overall delirium rate (33% pre versus 31% post; p = 0.84). However, there was a significant interaction between study phase and hospital (p = 0.03). Although one hospital did not experience a decline in delirium rate, the delirium rate at the other hospital declined from 42% to 19% (p = 0.08). This difference by hospital was mirrored in focus group feedback. The hospital that experienced a decline in delirium rates was more supportive of the intervention. Overall, post-intervention there were no significant differences in mean length of stay (12 days post versus 14 days pre; p = 0.74), falls (6% post versus 10% pre; p = 0.43) or discharges to long-term care (6% post versus 13% pre; p = 0.20). Translation of evidence-based multi-component delirium prevention strategies into everyday clinical care, using the electronic medical record, was not found to be effective at decreasing delirium rates among hip facture patients.

## Background

Delirium, or acute confusion, occurs in 25 to 65% of hospitalized patients treated for acute hip fracture [1,2]. Local data showed that orthopedic inpatients experienced the highest rates of delirium within the surgical subspecialties. Delirium is defined as an acute disturbance of consciousness accompanied by a change in cognition or by development of a perceptual disturbance [3]. Delirium develops over a short period of time, tends to fluctuate over time and is usually due to a general medical condition, substance intoxication, and/or substance withdrawal [3]. Hip fracture patients who develop delirium while in hospital have significantly worse outcomes than those who do not become delirious. Developing delirium in hospital has been associated with death, longer length of hospital stay, increased hospital-acquired complications, persistent cognitive deficits, and increased discharge rates to long-term care [4-7]. Delirium is also predictive of poor functional recovery among hip fracture patients [5,8].

There are a number of preoperative factors that increase the risk of developing delirium after surgery, including age, cognitive and functional impairment, alcohol abuse, depression, abnormal preoperative electrolytes, depression, co-morbid disease, sensory impairment, and residing in a care facility [9,10]. Several hospital-related precipitating factors also exist, including physical restraints, malnutrition and dehydration, urinary catheters, three or more new medications, and any iatrogenic event [11]. The cause of delirium is rarely due to just one factor; instead, multiple precipitating factors typically contribute to its development [11].

Prevention is a key strategy when addressing delirium, as after it occurs it can have devastating consequences [4-8]. Interventions do not clearly affect the duration of delirium once it develops [12]. Given that multiple factors usually contribute to the development of delirium, randomized trials have shown multi-component preventative strategies to be most effective [12-15]. However, given resource restraints, these multi-component strategies are not always easy to translate and implement into routine clinical care [16].

The evidence base around appropriate dissemination and implementation strategies is imperfect [17]. Computerized clinical decision support systems, which are information systems designed to improve clinical decision-making at the point of care, are one form of knowledge translation found to be effective [18-20]. The objective of this study was to determine if incorporation of an evidence-based multi-component delirium prevention strategy into an electronic post-operative hip fracture clinical pathway, which is a form of clinical decision support, would result in a decrease in delirium rates and related outcomes among older hip fracture patients.

## Methods

A pragmatic prospective cohort study using an interrupted time series design was conducted among patients admitted with a hip fracture to either of two Calgary, Alberta teaching hospitals [21]. All patients aged 65 years or older who were admitted for surgical repair of a hip fracture were eligible. Exclusion criteria included an inability to speak English, fractures caused by a motor vehicle crashes (given the mechanism of injury), or inability to consent to the study. Patients were monitored on the orthopedic wards for five months prior to implementation (October 2008 to March 2009) of the care pathway and then for five months post-implementation (March 2009 to August 2009).

**Figure 1:**
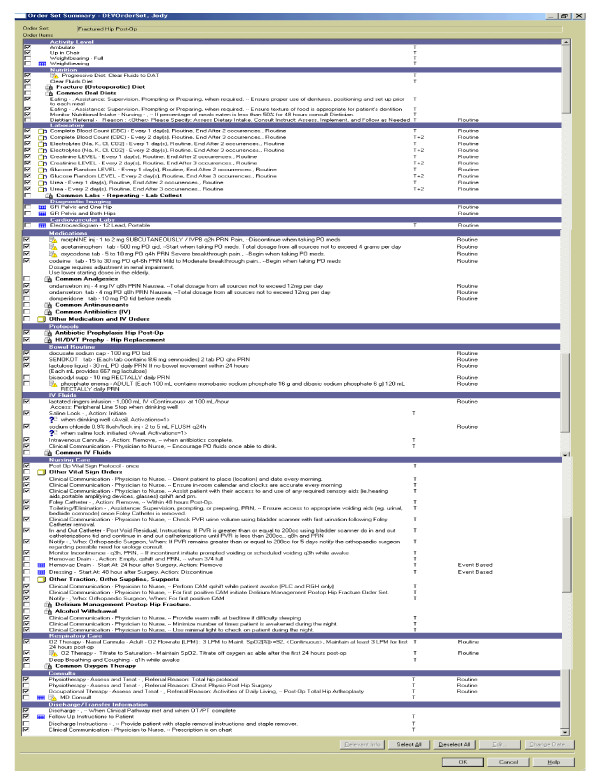
**Post-operative hip fracture order set with delirium prevention strategies**.

The care pathway was developed with input from information technologists, decision makers, researchers and frontline healthcare providers from orthopedics, geriatrics, and nursing. The delirium prevention strategies within the care pathway were based on evidence obtained from multi-component delirium prevention trials conducted in acute care settings [12-15]. The care pathway was developed to require minimal instruction for use in order to maximize adherence and sustainability within the dynamic work environment of a hospital orthopedic ward. The developed care pathway was embedded into the existing post-operative hip fracture order set located on the hospitals' electronic medical record (Figure 1).

The care pathway also incorporates the Confusion Assessment Method (CAM) [22], which is a brief delirium diagnostic tool that is accurate (sensitivity 86%, specificity 93%), with high interobserver reliability [23]. Prior to the study, the CAM was introduced on the orthopedic wards in order to aide in recognition of delirium. It was incorporated into the electronic post-operative hip fracture order set.

The primary study outcome was change in delirium rates as determined using a validated chart-based method for identification of delirium [24]. Secondary outcomes included length of hospital stay, in-hospital mortality, documented falls in hospital, new discharges to long-term care, and hospital readmission rates within 30 days. Data collection techniques were standardized and kept consistent throughout the study using an operations manual. One of two trained chart abstractors reviewed the hospital chart of each enrolled hip fracture patient admitted during any one of 40 separate weekly assessment time periods (20 pre- and 20 post-implementation). Based on annual local hip fracture admission rates of approximately 400 across the two hospitals, five months post-intervention surveillance (40 data points overall) was felt to be sufficient to detect uptake into practice [25]. Outcome data were collected from the hospital chart of enrolled patients up until their discharge from hospital or to the end of the 10-month study period. Readmissions to hospital were tracked for one month post-discharge. Patients were eligible for enrollment only once. A Durbin Watson test was conducted to test for serial correlation between weekly delirium rates and, because no correlation was found, Chi-Square and Wilcoxon tests were used to make univariable comparisons, while logistic regression analyses was used to compare the effect of phase on delirium rates, while adjusting for hospital. No other change to practice was known to have occurred during the study.

At study completion, focus groups (one at each hospital) were conducted with the frontline orthopedic nursing staff in order to explore issues around the implementation of the pathway. Nurses were recruited through postings and using snowball sampling. Participation was considered to be implied consent. Focus group participants were asked about facilitators and barriers to using the pathway, ease of use, and for specific feedback on its components. The focus groups were conducted, prior to analyzing the quantitative outcomes, by the principal investigator guided by a standardized list of questions. A research assistant took notes, which were later merged with data transcribed from audio recordings. The transcripts were coded by the investigator using a content analysis approach. Themes were identified and categorized. Only two focus groups were conducted due to limitations around the availability of frontline nurses to participate. This study received ethical approval from the University of Calgary Conjoint Health Research Ethics Board.

## Results

During the 40-week study period, 343 patients were potentially eligible for enrolment (173 pre- and 170 post-intervention). Among these patients 134 consented to participate, 21 declined participation, 138 were incapable of consenting, and 50 were determined to be otherwise ineligible. Among those enrolled, 102 were residing in their own home prior to their hip fracture.

**Figure 2:**
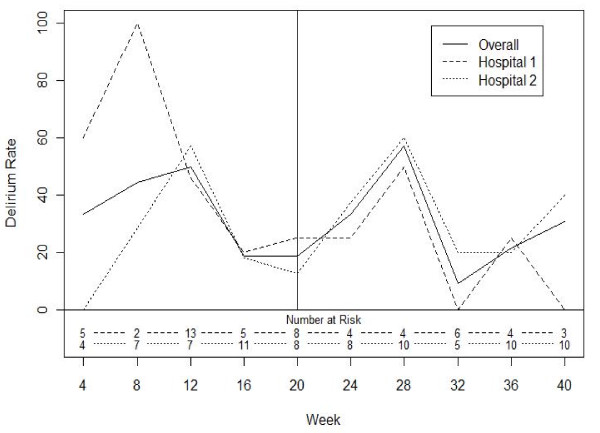
**Change in delirium rates over time overall and by hospital**.

The intervention had no effect on the overall delirium rate (Table 1). However, there was a significant interaction between study phase and hospital (p = 0.033). Although one hospital (hospital two) did not experience a decline in their delirium rate, the delirium rate at the other hospital (hospital one) declined from 42% to 19% with the intervention (p = 0.076; Figure 2). There were no significant changes in hospital length of stay, falls, or discharges to long-term care facilities (Table 1). There was one death among those enrolled, and six patients were readmitted to hospital (two pre- and four post-implementation; p = 0.340).

**Table 1:** Outcomes for the 134 hip fracture patients enrolled in the delirium prevention study

Outcome	Pre-intervention	Post-intervention	**Difference (95% CI)**^**a**^	**p value**^**b**^
Delirium, n/N (%)	23/70 (33)	20/64 (31)	2 (-14, 17)	0.840
Hospital 1, n/N (%)	14/33 (42)	4/21 (19)	23 (0, 47)	0.076
Hospital 2, n/N (%)	9/37 (24)	16/43 (37)	-13 (-33, 7)	0.220
Length of stay, median days (range)	14 (9-21)	12 (10-21)	-0.03 (-4.08, 4.03)	0.740
Hospital 1, median days (range)	14 (10-23)	11 (9-16)	-1.7 (-7.4, 4.0)	0.210
Hospital 2, median days (range)	14 (9-20)	13 (10-21)	2.9 (-2.9, 8.7)	0.630
Fall, n/N (%)	7/70 (10)	4/64 (6)	4 (-5, 13)	0.430
Hospital 1, n/N (%)	4/33 (12)	1/21 (5)	7 (-7, 22)	0.640
Hospital 2, n/N (%)	3/37 (8)	3/43 (7)	1 (-11, 13)	>0.99
New discharge to long-term care, n/N (%)	9/70 (13)	4/64 (6)	7 (-3, 16)	0.200
Hospital 1, n/N (%)	6/33 (18)	1/21 (5)	13 (-3, 29)	0.230
Hospital 2, n/N (%)	3/37 (8)	3/43 (7)	1 (-11, 13)	>0.99

^a^Estimated mean difference for continuous measures, estimated difference in proportions for categorical measures

^b^Wilcoxon p-value for continuous measures, chi-square or Fisher's exact test p-value for categorical measures

When focus group participants were asked about barriers to using the pathway, both groups felt that there was too much information to read and that orders could be missed. Although both focus groups felt the delirium strategies were based on 'common sense,' one group felt the orders were insulting and the overwhelming consensus amongst this group was that the pathway was 'painful' to use. The other focus group felt the delirium strategies were useful reminders of good practice, and all these participants felt the pathway was easy to use. Participants in this second group also commented that the pathway (and doing the CAM) helped them to identify delirium and initiate management strategies earlier. This second group was from the hospital that experienced a 50% reduction in delirium rates.

## Discussion

Our attempt to systematically incorporate evidence-based multi-component delirium prevention strategies [12-15] into practice resulted in mixed success. Although we made efforts to obtain input from all levels of the healthcare team during development, this project highlights the importance of continuing to engage frontline personnel because of issues like staff turnover and the development of unexpected barriers [26]. The focus groups highlighted the potential impact of organizational culture, personnel changes, and structure on the uptake of the delirium prevention strategies. Multiple factors can influence the uptake of evidence by different stakeholder groups with challenges operating at different levels within the system [26].

Effective knowledge translation includes adaptation of the intervention to address identified barriers [27]. Specifically, the order set was subsequently redesigned to address the concerns of the focus groups about the volume of information included. Although the content has not markedly changed, formatting changes have reduced the total number of orders. The modified intervention is informing a provincial hip fracture care pathway currently under development.

## Limitations

Although we used a validated chart-abstraction instrument, determining delirium rates was dependant on relevant information being recorded within the medical chart. Sample size calculations are challenging with interrupted time series studies [28]. We estimated that 40 data points would be sufficient to detect a change to practice [25]. However, we underestimated enrolment issues. Specifically, 138 patients were not enrolled due to issues around obtaining consent from patients. Extending the recruitment period was not feasible given funding limitations.

## Conclusions

Translation of evidence-based multi-component delirium prevention strategies into everyday clinical care, using an electronic health record, was not shown to be effective at decreasing delirium rates among hospitalized hip facture patients, although it was found to be clinically successful at one hospital. This project highlights the importance of end-user support when implementing evidence-based clinical decision support tools.

## Competing interests

The authors declare that they have no competing interests.

## Authors' contributions

All authors made substantial contributions to conception and design and to interpretation of data; FK analyzed the data; JH-L contributed to the acquisition of data and drafted the manuscript; All authors were involved in revising the manuscript critically for important intellectual content and have given final approval of the version to be published.

## References

[B1] Inouye SK Delirium after hip fracture: to be or not to be? J Am Geriatr Soc 2001 49 5 678 679 10.1046/j.1532-5415.2001.49133.x 11380767

[B2] Williams-Russo P Urquhart BL Sharrock NE Charlson ME Post-operative delirium: predictors and prognosis in elderly orthopedic patients J Am Geriatr Soc 1992 40 8 759 767 1634718 10.1111/j.1532-5415.1992.tb01846.x

[B3] DSM-IV-TR: Diagnostic and Statistical Manual of Mental Disorders 2000 4 The American Psychiatric Association

[B4] Francis J Kapoor WN Prognosis after hospital discharge of older medical patients with delirium J Am Geriatr Soc 1992 40 6 601 606 1587979 10.1111/j.1532-5415.1992.tb02111.x

[B5] Gustafson Y Berggren D Brannstrom B Bucht G Norberg A Hansson LI Acute confusional states in elderly patients treated for femoral neck fracture J Am Geriatr Soc 1988 36 6 525 530 2897391 10.1111/j.1532-5415.1988.tb04023.x

[B6] Inouye SK Rushing JT Foreman MD Palmer RM Pompei P Does delirium contribute to poor hospital outcomes? A three-site epidemiologic study J Gen Intern Med 1998 13 4 234 242 10.1046/j.1525-1497.1998.00073.x 9565386 PMC1496947

[B7] O'Keeffe S Lavan J The prognostic significance of delirium in older hospital patients J Am Geriatr Soc 1997 45 2 174 178 9033515 10.1111/j.1532-5415.1997.tb04503.x

[B8] Marcantonio ER Flacker JM Michaels M Resnick NM Delirium is independently associated with poor functional recovery after hip fracture J Am Geriatr Soc 2000 48 6 618 624 10855596 10.1111/j.1532-5415.2000.tb04718.x

[B9] Dasgupta M Dumbrell AC Preoperative risk assessment for delirium after noncardiac surgery: a systematic review J Am Geriatr Soc 2006 54 10 1578 1589 10.1111/j.1532-5415.2006.00893.x 17038078

[B10] Marcantonio ER Goldman L Mangione CM Ludwig LE Muraca B Haslauer CM A clinical prediction rule for delirium after elective noncardiac surgery JAMA 1994 271 2 134 139 10.1001/jama.271.2.134 8264068

[B11] Inouye SK Charpentier PA Precipitating factors for delirium in hospitalized elderly persons. Predictive model and interrelationship with baseline vulnerability JAMA 1996 275 11 852 857 10.1001/jama.275.11.852 8596223

[B12] Inouye SK Bogardus ST Jr Charpentier PA Leo-Summers L Acampora D Holford TR A multicomponent intervention to prevent delirium in hospitalized older patients N Engl J Med 1999 340 9 669 676 10.1056/NEJM199903043400901 10053175

[B13] Marcantonio ER Flacker JM Wright RJ Resnick NM Reducing delirium after hip fracture: a randomized trial J Am Geriatr Soc 2001 49 5 516 522 10.1046/j.1532-5415.2001.49108.x 11380742

[B14] Lundstrom M Olofsson B Stenvall M Karlsson S Nyberg L Englund U Postoperative delirium in old patients with femoral neck fracture: a randomized intervention study Aging Clin Exp Res 2007 19 3 178 186 17607084 10.1007/BF03324687

[B15] Vidan M Serra JA Moreno C Riquelme G Ortiz J Efficacy of a comprehensive geriatric intervention in older patients hospitalized for hip fracture: a randomized, controlled trial J Am Geriatr Soc 2005 53 9 1476 1482 10.1111/j.1532-5415.2005.53466.x 16137275

[B16] Reuben DB Making hospitals better places for sick older persons J Am Geriatr Soc 2000 48 12 1728 1729 11129768 10.1111/j.1532-5415.2000.tb03890.x

[B17] Grimshaw JM Thomas RE MacLennan G Fraser C Ramsay CR Vale L Effectiveness and efficiency of guideline dissemination and implementation strategies Health Technol Assess 2004 8 6 iii iv 1-72 14960256 10.3310/hta8060

[B18] Garg AX Adhikari NK McDonald H Rosas-Arellano MP Devereaux PJ Beyene J Effects of computerized clinical decision support systems on practitioner performance and patient outcomes: a systematic review Jama 2005 293 10 1223 1238 10.1001/jama.293.10.1223 15755945

[B19] Kawamoto K Houlihan CA Balas EA Lobach DF Improving clinical practice using clinical decision support systems: a systematic review of trials to identify features critical to success Bmj 2005 330 7494 765 10.1136/bmj.38398.500764.8F 15767266 PMC555881

[B20] Shojania KG Jennings A Mayhew A Ramsay CR Eccles MP Grimshaw J The effects of on-screen, point of care computer reminders on processes and outcomes of care Cochrane Database of Systematic Reviews 2009 3 Art. No.: CD001096 10.1002/14651858.CD001096.pub2 PMC4171964 19588323

[B21] Gottman J Time-series analysis 1981 New York: Cambridge University Press

[B22] Inouye SK van Dyck CH Alessi CA Balkin S Siegal AP Horwitz RI Clarifying confusion: the confusion assessment method. A new method for detection of delirium Ann Intern Med 1990 113 12 941 948 2240918 10.7326/0003-4819-113-12-941

[B23] Wong CL Holroyd-Leduc J Simel DL Straus SE Does this patient have delirium? Value of bedside instruments JAMA 2010 304 7 779 786 10.1001/jama.2010.1182 20716741

[B24] Inouye SK Leo-Summers L Zhang Y Bogardus ST Jr Leslie DL Agostini JV A chart-based method for identification of delirium: validation compared with interviewer ratings using the confusion assessment method J Am Geriatr Soc 2005 53 2 312 318 10.1111/j.1532-5415.2005.53120.x 15673358

[B25] Cook TD Campbell DT Quasi-experiments: interrupted time-series designs in quasi-experimentation: design and analysis issues for field settings 1979 Boston: Houghton Mifflin Company

[B26] Straus SE Tetroe J Graham I Defining knowledge translation CMAJ 2009 181 3-4 165 168 19620273 10.1503/cmaj.081229 PMC2717660

[B27] Graham ID Logan J Harrison MB Straus SE Tetroe J Caswell W Lost in knowledge translation: time for a map? J Contin Educ Health Prof 2006 26 1 13 24 10.1002/chp.47 16557505

[B28] Ramsay CR Matowe L Grilli R Grimshaw JM Thomas RE Interrupted time series designs in health technology assessment: lessons from two systematic reviews of behavior change strategies Int J Technol Assess Health Care 2003 19 4 613 623 10.1017/S0266462303000576 15095767

